# Genetic evidence supporting the causal role of 25-hydroxyvitamin D levels in the prognosis of ER− breast cancer: A Mendelian randomization study

**DOI:** 10.1097/MD.0000000000040262

**Published:** 2024-10-25

**Authors:** Shang Wu, Xin-Di Ma, Xiang-Mei Zhang, Chao Shi, Kai-Ye Du, Yun-Jiang Liu

**Affiliations:** a Breast Center, The Fourth Hospital of Hebei Medical University, Shijiazhuang, China; b Hebei Provincial Key Laboratory of Tumor Microenvironment and Drug Resistance, Hebei Medical University, Shijiazhuang, China; c Research Center, The Fourth Hospital of Hebei Medical University, Shijiazhuang, China; d Radiotherapy Department, The Fourth Hospital of Hebei Medical University, Shijiazhuang, China.

**Keywords:** breast cancer, estrogen receptor, genetics, 25-hydroxyvitamin D, Mendelian randomization study

## Abstract

This study aims to investigate the connection between 25-hydroxyvitamin D (25(OH)D) levels and the prognosis of breast cancer with various estrogen receptor (ER) statuses. The summary statistics of 25(OH)D levels was obtained from a GWAS of 441,291 individuals and the information of breast cancer was collected from the Breast Cancer Association Consortium. We analyzed the causal association between 25(OH)D levels and breast cancer prognosis using a number of approaches, including inverse variance weighting (IVW). The heterogeneity test was performed using Cochran Q test. IVW, Mendelian randomization (MR)-Egger, and MR Pleiotropy RESidual Sum and Outlier methods were used for sensitivity analysis. In addition, a multivariate MR adjusted for total triglycerides, total cholesterol, and body mass index was used for further analysis. Two-sample MR results showed that 25(OH)D levels were not associated with prognosis in overall breast cancer (odds ratio [OR] = 0.93, 95% confidence interval [CI] = 0.73–1.19, IVW exam) and estrogen receptor positive (ER+) breast cancers (OR = 1.12, 95% CI = 0.77–1.63, IVW exam) and were protective associated with prognosis in estrogen receptor negative (ER−) breast cancers (OR = 0.55, 95% CI = 0.34–0.87, IVW exam). Sensitivity analysis did not observe the presence of heterogeneity and horizontal pleiotropy. In multivariate MR analysis, after adjusting for total triglycerides, total cholesterol, and body mass index, the correlation between the protective relationship between 25(OH)D levels and the prognosis for ER− breast cancer remained and became increasingly significant (OR = 0.51, 95% CI = 0.31–0.83, *P* = .007). This study demonstrated a protective relationship between 25(OH)D levels and the prognosis of ER− breast cancer, but there was no connection between 25(OH)D levels and the prognosis of ER+ breast cancer.

## 1. Introduction

As the most frequent cancer among women diagnosed worldwide, breast cancer has overtaken lung cancer. There will be 690,000 fatalities from breast cancer and 2.3 million new cases globally in 2020, putting women’s physical and mental health at significant danger.^[1,2]^ Due to medication resistance and metastasis, some breast cancer patients still have unfavorable prognoses despite the fast advancements in treatment.^[3,4]^ Therefore, additional predictors are required to estimate the outcome of various breast cancer patients and assist patients in developing individualized breast cancer treatment plans.^[5,6]^

Serum levels of 25-hydroxyvitamin D (25(OH)D), a hormone precursor that regulates calcium homeostasis, are thought to be the most accurate measure of vitamin D status.^[7]^ Not only does vitamin D have effects on bones, but its various anti-cancer effects are being discovered,^[8,9]^ and these findings provide theoretical support for the association between vitamin D and cancer incidence and survival.^[10]^ Previous studies have shown that the active component of vitamin D affects estrogenic or non-estrogenic signaling mechanisms that trigger apoptosis in breast cancer cells.^[11,12]^ High 25(OH)D levels may eliminate or decrease the amount of breast cancer cells in the body. Therefore, increasing interest in the influence of 25(OH)D levels on the occurrence and outcome of breast cancer has attracted a growing amount of focus.^[13–15]^ But studies on 25(OH)D levels and breast cancer prognosis have reached inconsistent conclusions from different studies. Some studies have found that high serum 25(OH)D levels at the time of breast cancer diagnosis are associated with a better prognosis,^[16–21]^ while other investigations reveal an absence of relationship between serum 25(OH)D levels and patient prognosis.^[22,23]^ Additionally, it has been found that postmenopausal individuals with high levels of 25(OH)D had higher overall survival and breast cancer-specific survival than premenopausal patients.^[24]^

Due to multiple invisible confounding variables, reverse causality, and bias, traditional observational research may provide skewed data or contradictory conclusions. Using genetic variations known as single nucleotide polymorphisms (SNPs) as instrumental variables (IVs) to deduce causal links between exposures and outcomes, Mendelian randomization (MR) is a novel genetics-based research tool.^[25]^ Since SNPs are randomly assigned to offspring during cell meiosis to fertilized egg formation, genetic variants precede disease onset, which eliminates the effect of reverse causation.^[26]^ Negative results have occurred due to the possible poor design of previous randomized controlled studies of the relationship between 25(OH)D levels and cancers and the large variability in 25(OH)D levels concentrations due to the long follow-up time in some cohort studies.^[27,28]^ Consequently, MR is the perfect instrument to investigate the causal connection between 25(OH)D levels and the long-term outcome of breast cancer. Previous research has shown that various risk factors affect breast cancer differently depending on the estrogen level,^[29]^ so this study combined two-sample MR and multiple-sample MR analysis to investigate whether there is a causal association between 25(OH)D levels and the outcome of breast cancer with varying estrogen receptor (ER) status, to evaluate its impact, and to develop a more individualized treatment for patients.

## 2. Material and methods

### 2.1. Data collection

In the current study, 25(OH)D levels and breast cancer (survival) served as the exposure and outcome, respectively. SNPs associated with 25(OH)D levels (inquiry code: ieu-b-4812) were collected from the MRCIEU GWAS database (https://gwas.mrcieu.ac.uk/). The 25(OH)D levels database contained 441,291 samples, including 16,668,957 SNPs. The Breast Cancer Association Consortium provided information regarding SNPs associated with breast cancer survival in varying ER states.^[30]^ Of these, overall breast cancer (inquiry code: ieu-a-1165) contained 37,954 samples which containing 12,940,150 SNPs. Estrogen receptor positive (ER+) breast cancer (inquiry code: ieu-a-1164) included 23,059 samples and 8714,606 SNPs, whereas estrogen receptor negative (ER−) breast cancer (inquiry code: ieu-a-1163) included 6881 samples and 8,828,662 SNPs. All information originated with the people of Europe. Our paper did not need to go through an ethical review since it was based on data that was already in public databases.

### 2.2. Instrumental variable extraction

Meeting the 3 MR hypotheses is necessary for the selection of SNPs as IVs to evaluate the causal effect of 25(OH)D levels on breast cancer prognosis: (1) there must be a considerable correlation between genetic variations and 25(OH)D levels. (2) Confounding factors had no effect on the IVs that determine the relationship between 25(OH)D levels and breast cancer prognosis. (3) IVs can only affect breast cancer survival through 25(OH)D levels. The standards for screening SNPs are as follows: (i) there were substantial connections between SNPs and 25(OH)D levels (*P* < 5 × 10^−8^), and the obtained SNPs were then used to represent vitamin 25(OH)D levels. (ii) To prevent bias brought on by linkage disequilibrium, SNPs are independent of one another (*r*^2^ < 0.001). (iii) The term “genetic distance” refers to the distance’s length (LD), and we used an LD of 10,000 kb. SNPs with *r*^2^ over 0.001 and LD < 10,000 kb will be removed. Additionally eliminated were palindromic SNPs with intermediate allele frequencies. Also, we checked on the PhenoScanner website (http://www.phenoscanner.medschl.cam.ac.uk/) to identity the IVs included in this study, and if SNPs were associated with the breast cancer survival, they would be removed prior to MR analysis. Additionally, we tested the potential pleiotropy of the chosen SNPs using MR Pleiotropy RESidual Sum and Outlier (MR-PRESSO), and the findings revealed that no SNPs were eliminated.

### 2.3. Two-sample MR analysis

The correlation between 25(OH)D levels and the prognosis of breast cancer is first examined using two-sample MR. Ninety-nine SNPs were shown to be associated with overall breast cancer survival, ER+ breast cancer survival, and ER− breast cancer survival in the two-sample MR study’s final findings. Tables S1 to S3, Supplemental Digital Content, http://links.lww.com/MD/N812, present the original data. We largely employed inverse variance weighting (IVW) to aggregate the results of numerous separate research, which is a technique for minimizing the sum variance by combining 2 or more random variables, where the variance of each random variable is inversely correlated with its weight in the aggregate.^[31]^ To guarantee that the outcomes are accurate, various methods including MR-Egger, simple model, weighted model, and maximum likelihood were used to further validate the results.

### 2.4. Sensitivity analysis

To determine whether genetic pleiotropy existed or not, we employed the intercept of the MR-Egger regression model. The impact of genetic pleiotropy was deemed to not exist if the intercept term’s *P* value was larger than .05. Then, we acknowledged that IVs could only influence breast cancer survival through 25(OH)D levels. The heterogeneity of IVs was evaluated by applying the Cochran Q test. We then plotted funnel plots to further analyze whether there was heterogeneity in the results. Also, we used leave-one-out analysis to remove each SNP one by one, and the remaining SNPs continued MR analysis to examine the sensitivity of the results. The MR-PRESSO test was performed to determine if the findings of the MR analysis before and after correction were different.

### 2.5. Multifactorial MR analysis

We investigated the PhenoScanner website (http://www.phenoscanner.medschl.cam.ac.uk/) for 99 SNPs obtained from two-sample MR analysis, and some of these SNPs were associated with total cholesterol, total triglycerides, and body mass index (BMI). Previous MR studies demonstrated associations between instruments and total cholesterol, total triglycerides, and BMI characteristics.^[32–34]^ To avoid confounding effects, we used a multifactorial MR and added total cholesterol, total triglycerides, and BMI to adjust for the causal relationship between 25(OH)D levels and outcomes. Therefore, we selected SNPs for total cholesterol from a GWAS of 115,078 individuals (inquiry code: met-d-Total_C), SNPs for total triglycerides from a GWAS of 115,078 individuals (inquiry code: met-d-Total_TG), and SNPs for BMI from a GWAS of 461,460 individuals (inquiry code: ukb-b-19953) for the multivariable MR analyses. These are accessible through the MRCIEU GWAS database (https://gwas.mrcieu.ac.uk/).

### 2.6. Statistical analysis

The statistical software R (version 4.2.2) was applied for all analyses, and the “TwoSampleMR” R package was used to carry out the MR correlation study as well as the sensitivity analysis. Odds ratio (OR) with associated 95% confidence interval (CI) were used to represent the outcomes of the two-sample and multivariate MR analyses. Differences that were statistically significant were those with two-sided *P* values below .05.

## 3. Results

### 3.1. Two sample MR analysis

**Figure 1.**
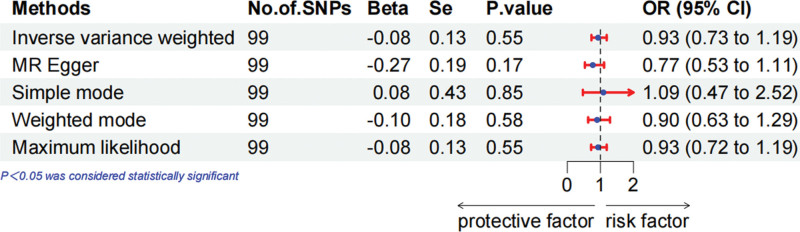
Mendelian randomization approach to explore the effect of 25-hydroxyvitamin D levels on the prognosis of overall breast cancer in forest plots.

**Figure 2.**
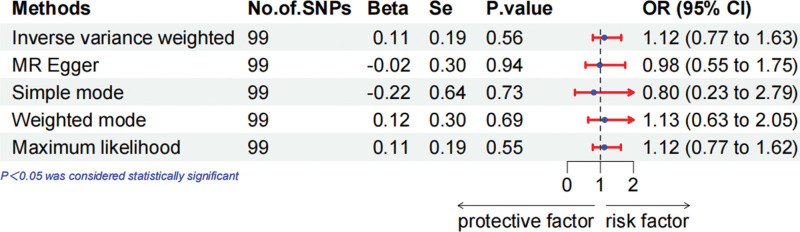
Mendelian randomization approach to explore the effect of 25-hydroxyvitamin D levels on the prognosis of estrogen receptor positive breast cancer in forest plots.

**Figure 3.**
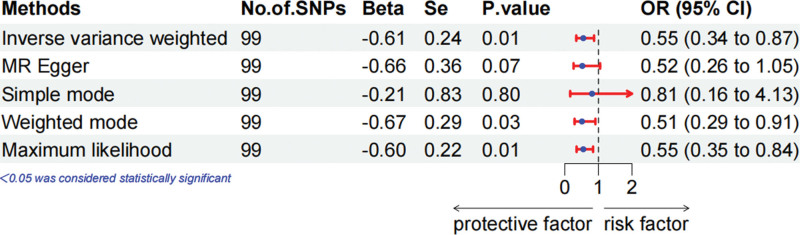
Mendelian randomization approach to explore the effect of 25-hydroxyvitamin D levels on the prognosis of estrogen receptor-negative breast cancer in forest plots.

MR analysis demonstrated that 25(OH)D levels were unrelated to the overall breast cancer prognosis (Fig. 1, OR = 0.93, 95% CI = 0.73–1.19, IVW exam). When we performed a subgroup analysis based on ER status, in ER+ breast cancer, we observed that 25(OH)D levels did not substantially correlate with survival (Fig. 2, OR = 1.12, 95% CI = 0.77–1.63, IVW exam), but protective correlated with survival in ER− breast cancer (Fig. 3, OR = 0.55, 95% CI = 0.34–0.87, IVW exam). To guarantee that the outcomes are accurate, other methods were used to assess the correlation. The weighted mode and maximum likelihood methods also showed that 25(OH)D levels were protective factors for ER− breast cancer survival.

### 3.2. Sensitivity analysis

The outcomes of the sensitivity analysis we carried out further support the validity of our findings (Table 1). Regarding overall breast cancer survival, neither the IVW exam (Q = 97, *P* = .51) nor the MR-Egger exam (Q = 95, *P* = .53) revealed any heterogeneity. Additionally, ER− and ER+ breast cancer survivorship did not exhibit any discernible heterogeneity (Table 1). The funnel plot further visualizes the results with a uniform scatter distribution, showing no heterogeneity in the results (Figure S1, Supplemental Digital Content, http://links.lww.com/MD/N811). According to the outcomes of the MR-Egger intercept test, there is no horizontal pleiotropy between exposures and outcomes (*P* ＞ .05). The MR-PRESSO test also did not detect pleiotropy, ensuring the accuracy of the results. The scatter plots of MR analyses are shown in Figure S2, Supplemental Digital Content, http://links.lww.com/MD/N811. Furthermore, the findings of the leave-one-out analysis also revealed that there was no significant impact of SNP alone on the outcome for both overall breast cancer survival and ER+ breast cancer survival. For ER− breast cancer survival, leave-one-out analysis showed statistically significant results regardless of which SNP was removed alone, confirming the validity of our findings and revealing a protective relationship between 25(OH)D levels and survival in ER− breast cancer (Figures S3–S5, Supplemental Digital Content, http://links.lww.com/MD/N811).

**Table 1:** Sensitivity analysis of Mendelian randomization of the causal relationship between 25-hydroxyvitamin D levels and prognosis of breast cancer with different estrogen receptor status.

	Heterogeneity				Pleiotropy		Outlier examination by MR-PRESSO					
	MR-Egger		IVW		MR-Egger		Before correction			After correction (if necessary)		
	Q	*P* value	Q	*P* value	Intercept	*P* value	MR analysis causal estimate	SD	*P* value	MR analysis causal estimate	SD	*P* value
Overall	95	.53	97	.51	0.007	.19	-0.08	0.13	.55	NA	NA	NA
ER+	103	.33	103	.34	0.005	.55	0.11	0.19	.57	NA	NA	NA
ER−	116	.10	116	.11	0.002	.84	-0.61	0.24	.01	NA	NA	NA

ER+ = estrogen receptor positive, ER- = estrogen receptor negative, IVW = inverse variance weighted, MR = Mendelian randomization, MR-PRESSO = MR Pleiotropy RESidual Sum and Outlier.

### 3.3. Multivariate MR analysis

Multivariate MR analysis demonstrated that 25(OH)D levels were unrelated to both overall breast cancer survival (OR = 0.80, 95% CI = 0.61–1.06, *P* = .12) and ER+ breast cancer survival after adjusting for total triglycerides, total cholesterol, and BMI (OR = 0.87, 95% CI = 0.56–1.34, *P* = .53) (Table 2). Interestingly, after adjustment, the connection between 25(OH)D levels and ER− breast cancer survival was stronger (OR = 0.51, 95% CI = 0.31–0.83, *P* = .007) (Table 2), further demonstrating the accuracy of our results.

**Table 2:** Multivariable MR in breast cancer survival with different status of estrogen receptor.

	No. of SNPs	Beta	SE	OR (95% CI)	*P* value
Overall breast cancer					
25-Hydroxyvitamin D level	47	-0.22	0.14	0.80 (0.61–1.06)	.12
Total cholesterol	31	-0.19	0.12	0.83 (0.65–1.05)	.11
Total triglycerides	35	0.02	0.09	1.02 (0.86–1.22)	.79
BMI	332	0.01	0.10	1.01 (0.83–1.23)	.94
ER+ breast cancer					
25-Hydroxyvitamin D level	47	-0.14	0.22	0.87 (0.56–1.34)	.53
Total cholesterol	30	-0.14	0.18	0.87 (0.61–1.24)	.44
Total triglycerides	35	-0.04	0.13	0.96 (0.74–1.24)	.76
BMI	331	0.06	0.16	1.06 (0.78–1.45)	.72
ER- breast cancer					
25-Hydroxyvitamin D level	47	-0.68	0.25	0.51 (0.31–0.83)	.007
Total cholesterol	31	-0.25	0.21	0.78 (0.52–1.18)	.23
Total triglycerides	34	0.04	0.16	1.04 (0.76–1.42)	.81
BMI	331	-0.20	0.19	0.82 (0.56–1.19)	.28

BMI = body mass index, CI = confidence interval, ER+ = estrogen receptor positive, ER- = estrogen receptor negative, MR = Mendelian randomization, OR = odds ratio, SNPs = single nucleotide polymorphisms.

## 4. Discussion

Our two-sample MR analysis demonstrated that whereas 25(OH)D levels were not substantially linked with the prognosis of ER+ breast cancer, they were connected with ER− breast cancer prognosis, indicating protective association. This protective correlation in ER− breast cancer survival maintained after adjusting for total cholesterol, total triglycerides, and BMI, and the correlation became even more significant. Previous research conducted have demonstrated a causal relationship between BMI and triple-negative breast cancer.^[35]^ However, the connection only applies to incidence rather than prognosis. Therefore, further study is required to ascertain how BMI influences the prognosis of ER− breast cancer.

Vitamin D is a prevalent vitamin that is fat-soluble.^[36]^ 25(OH)D is a widely utilized biomarker of vitamin D levels, and there has been considerable interest in the role of 25(OH)D in non-calcium functions over the past few years. Several studies have discovered that serum 25(OH)D levels are connected to a wide range of diseases, which involves many malignancies.^[37]^ 25(OH)D levels have been predictive of breast cancer patients’ prognoses, according to a number of studies. In a meta-analysis of prospective cohort studies involving 4413 patients with breast cancer, higher levels of 25(OH)D (>75 nmol/L) were found to be associated with a decreased likelihood of mortality.^[38]^ Although this study establishes the optimal 25(OH)D cutoff for precise evaluations, it was unable to further categorize hormone receptor status. Our findings are an extension of their findings. A lower concentration of 25(OH)D were associated with a worse outcome in breast cancer patients, according to a recent meta-analysis that included 12 trials and 8574 breast cancer patients.^[39]^ The survival outcomes here were more specific and included overall survival, breast cancer-specific survival, and disease-free survival. This study included a larger number of patient cases, but the studies in this one included retrospective analyses that may have introduced confounding factors, and still no stratified analysis of ER status was performed either. Serum 25(OH)D deficit following the conclusion of 5 years of endocrine therapy was related to worse recurrence-free survival in ER+ breast cancer, according to a regression analysis conducted in Korea with 455 patients.^[40]^ This contrasts with what we found in our research results, which showed that 25(OH)D was exclusively related to survival in ER- breast cancer rather than in ER+ breast cancer. However, it has also been demonstrated that 25(OH)D can synergistically inhibit a number of proteins essential to the survival of triple-negative breast cancer cells, and that patients with the triple-negative phenotype have reduced 25(OH)D levels,^[41]^ and that patients with the triple-negative phenotype have reduced 25(OH)D levels,^[42]^ which indirectly supports our conclusion. Previous research has demonstrated that serum 25(OH)D levels fluctuate over time, including alterations following breast cancer being diagnosed.^[43]^ The 25(OH)D levels in patients prior to and after chemotherapy therapy have been shown to fall during chemotherapy but to increase within 6 months of chemotherapy to levels seen in women without carcinoma.^[44]^ These studies do show how temporal patterns and reverse causality of exposure could bias judgments.^[43]^ This clarifies the reason why some retrospective investigations came to the conclusion that 25(OH)D levels are not related to the prognosis of breast cancer^[23]^ or that those diagnosed with greater levels of 25(OH)D still had a worse prognosis.^[45]^

It is uncertain how 25(OH)D levels relate to the prognosis for breast cancer. According to preliminary research, vitamin D’s active ingredient may prevent breast cancer tumor cells from proliferating by blocking a certain cell cycle.^[46]^ Meanwhile, mechanistic investigations have revealed that the potent form of vitamin D influences the production of the enzyme cyclooxygenase 2, the NF-κB pathway, and the cytokines, contributing to the advancement of malignancy.^[47]^ The vitamin D receptor signaling pathway can also exert an autonomous inhibitory function on breast cancer tumor cell metastasis through the regulation of inhibitor of differentiation 1.^[48]^ According to a study, vitamin D’s active form, known as 25(OH)D, can cause apoptosis through a mechanism unrelated to estrogen signaling. This discovery highlights the potential that vitamin D can be used to treat ER− breast cancer, which also provides a mechanistic explanation for our findings that high concentrations of 25(OH)D protect against ER− breast cancer.^[11]^ In the meanwhile, vitamin D influences the immunological and neurological systems and may control a number of biological processes, including cell development and differentiation.^[49,50]^ It has reportedly been connected with several kinds of mental and neurological conditions.^[51,52]^ In certain breast cancer patients with inadequate 25(OH)D degrees, their health-related standard of life is diminished, as seen by higher levels of felt stress, weariness, and depression, which may be linked to the control of by 25(OH)D.^[53]^ By impacting breast cancer patients’ quality of life, this may indirectly alter their prognosis.

This research offers a number of advantages. It is the first research that investigates the relationship between the degrees of 25(OH)D and breast cancer survival using a MR strategy. This research was able to reduce possible confounders and reverse causation by pooling a significant quantity of genetic data. Second, we used a multifactorial MR approach to correct 25(OH)D levels for total triglycerides, total cholesterol, and BMI, thus making our results more accurate. Third, a subgroup analysis was performed determined by ER status and identified that only in ER− breast cancer did 25(OH)D levels functioned as a protective factor for survival. This can provide clinical advice on treatment and develop a more appropriate treatment for patients.

However, there are some limitations in our research. First, the participants in this study were from populations in the European region, and there are limitations in extending the findings to populations in other regions. Indeed, previous research has demonstrated that the ER− breast cancer incidence caused by genetic variations in the vitamin D pathway varies across populations.^[54–56]^ Second, this study’s sample size was modest. If possible, we will continue to conduct studies with larger sample sizes. Third, the mechanism behind the prognostic impact of the levels of 25(OH)D in improving ER− breast cancer cannot be clarified through MR techniques, which can only be employed for the investigation of causality. To figure out the mechanism by which 25(OH)D levels influence breast cancer prognosis, additional experimental investigations are required. Fourth, we were unable to further determine the ideal 25(OH)D cutoff value for accurately classifying high- and low-risk patients in order to direct the future therapy.

## 5. Conclusion

This study shows a protective correlation between 25(OH)D levels and the prognosis of ER− breast cancer, however, there is no significant correlation between 25(OH)D levels and the ER+ breast cancer survival. These findings suggest that higher 25(OH)D levels predict a better prognosis of ER− breast cancer.

## Author contributions

**Conceptualization:** Shang Wu, Xin-Di Ma, Xiang-Mei Zhang, Yun-Jiang Liu.

**Data curation:** Shang Wu, Xin-Di Ma, Xiang-Mei Zhang, Chao Shi, Kai-Ye Du.

**Formal analysis:** Shang Wu, Xin-Di Ma, Xiang-Mei Zhang, Chao Shi, Kai-Ye Du.

**Funding acquisition:** Yun-jiang Liu.

**Investigation:** Shang Wu, Xin-Di Ma, Xiang-Mei Zhang, Chao Shi.

**Methodology:** Shang Wu, Xin-Di Ma, Xiang-Mei Zhang, Chao Shi.

**Project administration:** Shang Wu, Xin-Di Ma, Xiang-Mei Zhang, Chao Shi, Kai-Ye Du.

**Resources:** Shang Wu, Xin-Di Ma, Xiang-Mei Zhang, Chao Shi, Kai-Ye Du.

**Software:** Shang Wu, Xin-Di Ma, Xiang-Mei Zhang.

**Supervision:** Shang Wu, Xin-Di Ma, Xiang-Mei Zhang.

**Validation:** Shang Wu, Xin-Di Ma, Xiang-Mei Zhang, Yun-Jiang Liu.

**Visualization:** Shang Wu, Xin-Di Ma, Xiang-Mei Zhang, Yun-Jiang Liu.

**Writing – original draft:** Shang Wu, Xin-Di Ma.

**Writing – review & editing:** Yun-Jiang Liu.

## Supplementary Material


