# Supplementary material for: Genetic evidence supporting the causal role of 25-hydroxyvitamin D levels in the prognosis of ER− breast cancer: A Mendelian randomization study
Source: Medicine (Baltimore). 2024 Oct 25;103(43):e40262. doi: 10.1097/MD.0000000000040262 (PMC11521050; doi:10.1097/MD.0000000000040262)
Supplement: Supplementary file 2 [file medi-103-e40262-s002.docx]

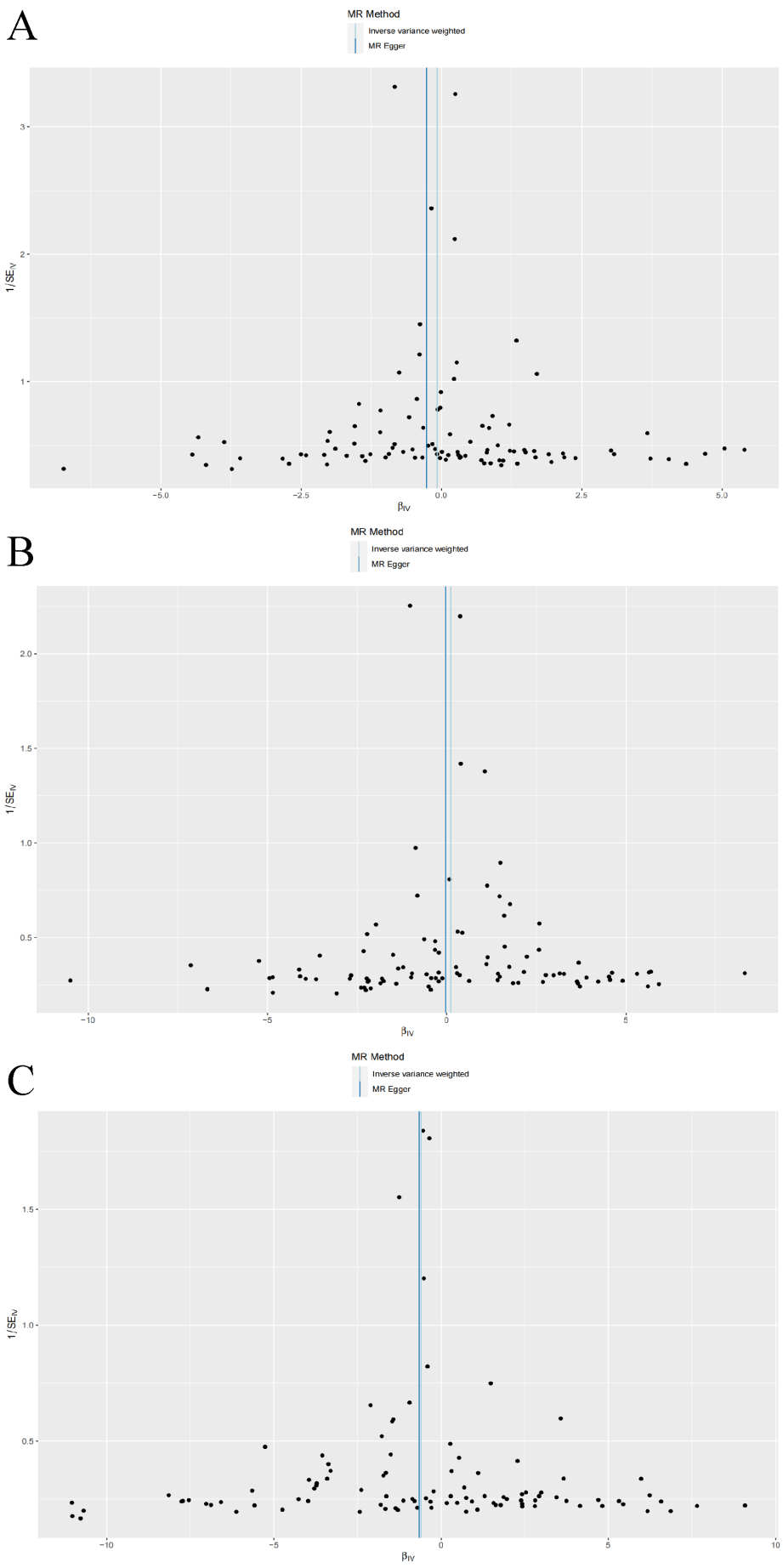


Supplement figure 1. Funnel plot of SNPs associated with 25 hydroxyvitamin D levels and the prognosis of (A) total breast cancer, (B) ER+ breast cancer, (C) ER- breast cancer.


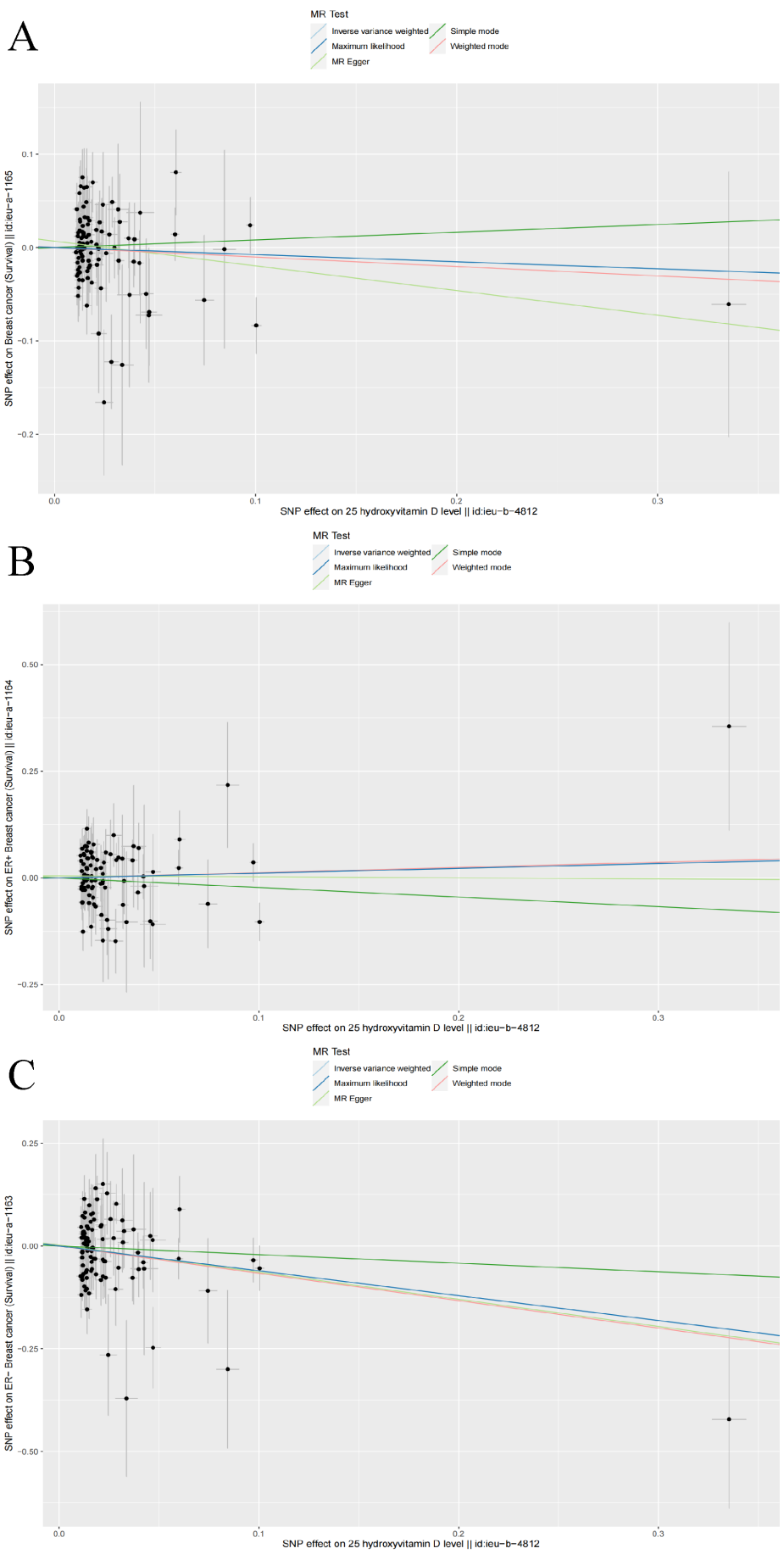


Supplement figure 2. Scatter plot of SNPs associated with 25 hydroxyvitamin D levels and the prognosis of (A) total breast cancer, (B) ER+ breast cancer, (C) ER- breast cancer.


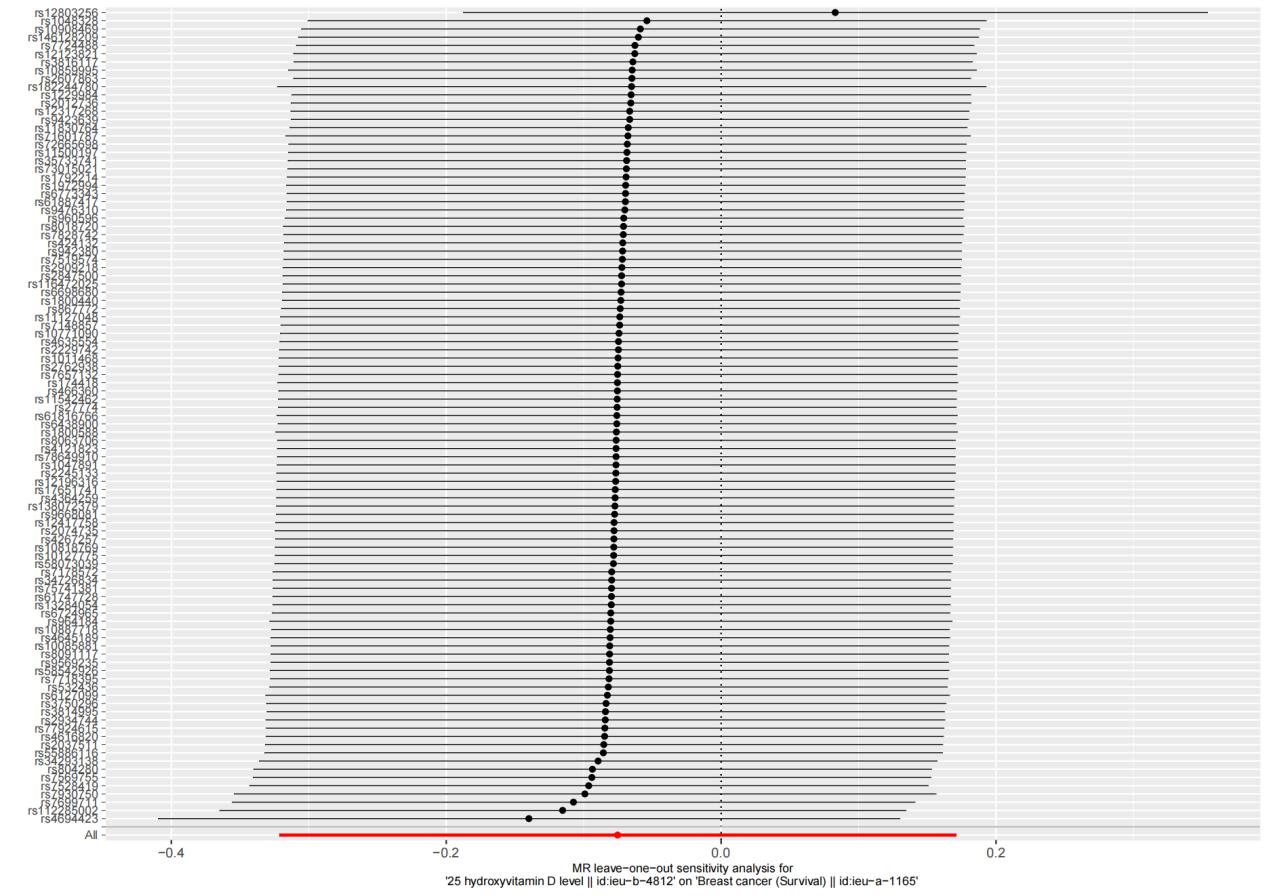


Supplement figure 3. Leave-one-out analysis result of SNPs associated with 25 hydroxyvitamin D levels and the prognosis of total breast cancer.


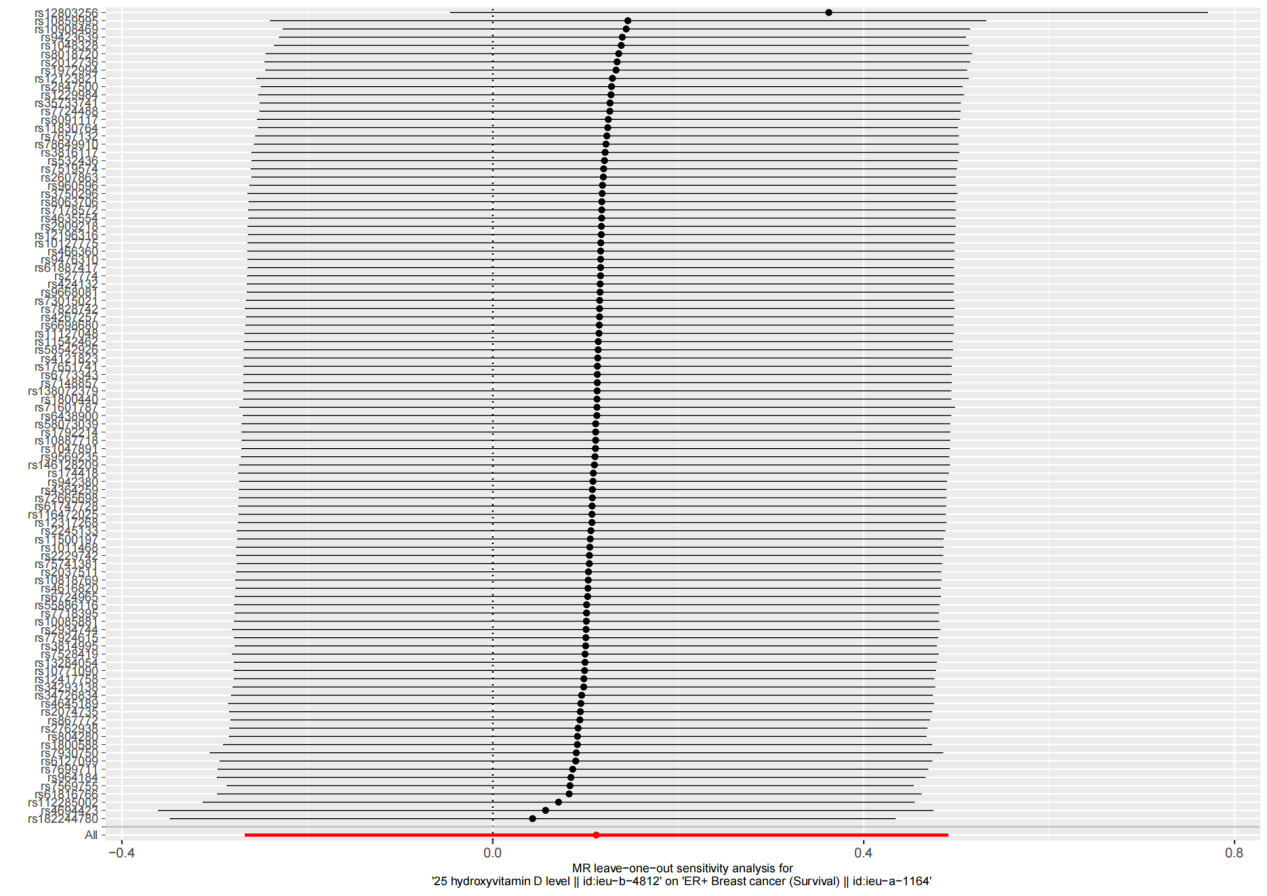


Supplement figure 4. Leave-one-out analysis result of SNPs associated with 25 hydroxyvitamin D levels and the prognosis of estrogen receptor positive breast cancer.


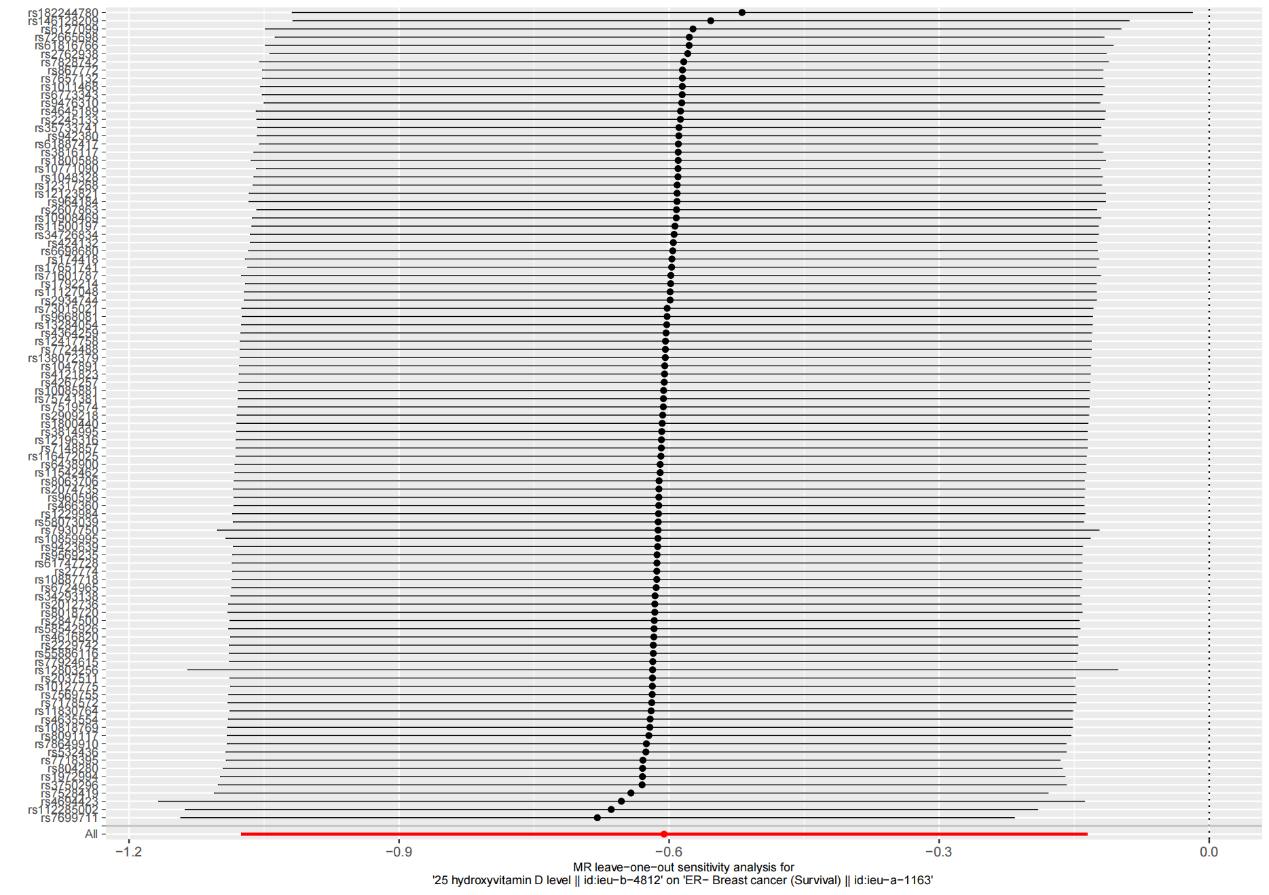


Supplement figure 5. Leave-one-out analysis result of SNPs associated with 25 hydroxyvitamin D levels and the prognosis of estrogen receptor negative breast cancer.
